# Ultra-Wideband Passive Polarization Conversion Metasurface for Radar Cross-Section Reduction Across C-, X-, Ku-, and K-Bands

**DOI:** 10.3390/mi16030292

**Published:** 2025-02-28

**Authors:** Xiaole Ren, Yunqing Liu, Zhonghang Ji, Qiong Zhang, Wei Cao

**Affiliations:** 1 College of Electronic Information Engineering, Changchun University of Science and Technology, Changchun 130022, China; ren@mails.cust.edu.cn (X.R.); zhonghang.ji@cust.edu.cn (Z.J.); zhangqiong15@mails.jlu.edu.cn (Q.Z.); w.cao@mails.cust.edu.cn (W.C.); 2 Jilin Provincial Science and Technology Innovation Center of Intelligent Perception and Information Processing, Changchun 130022, China

**Keywords:** passive anisotropic metasurface, polarization conversion, ultra-wideband, radar cross-section reduction, C-, X-, Ku- and K-bands

## Abstract

In this study, we present a novel ultra-wideband passive polarization conversion metasurface (PCM) that integrates double V-shaped patterns with circular split-ring resonators. Operating without any external power supply or active components, this design effectively manipulates the polarization state of incident electromagnetic waves. Numerical and experimental results demonstrate that the proposed PCM can convert incident linear polarization into orthogonal states across a wide frequency range of 7.1–22.3 GHz, encompassing the C-, X-, Ku-, and K-bands. A fabricated prototype confirms that the polarization conversion ratio (PCR) exceeds 90% throughout the specified band. Furthermore, we explore an additional application of this passive metasurface for electromagnetic stealth, wherein it achieves over 10 dB of monostatic radar cross-section (RCS) reduction from 7.6 to 21.5 GHz. This broad effectiveness is attributed to strong electromagnetic resonances between the top and bottom layers, as well as the Fabry–Pérot cavity effect, as evidenced by detailed analyses of the underlying physical mechanisms and induced surface currents. These findings confirm the effectiveness of the proposed design and highlight its potential for future technological applications, including 6G communications, radar imaging, anti-interference measures, and electromagnetic stealth.

## 1. Introduction

Metasurfaces, composed of periodic or aperiodic two-dimensional planar metamaterials, are innovative artificial electromagnetic materials that offer flexible layouts, ease of fabrication, and compatibility with integrated circuits. By leveraging specially designed or adjusted unit cells, metasurfaces can control the amplitude, phase, and polarization of electromagnetic waves, rapidly attracting extensive attention in the radio frequency domain [1]. Among these functionalities, polarization conversion, a key technique in electromagnetic wave manipulation, is employed in a broad spectrum of domains, such as wireless communication systems [2,3,4], imaging techniques featuring ultra-high resolution [5,6], chiral sensing [7], holography [8,9], optical multiplexing [10], and methods aimed at reducing radar cross-section (RCS) [11]. Compared to traditional polarization conversion devices, anisotropic metasurface polarization converters feature more flexible designs and higher conversion efficiencies and are more amenable to mass manufacturing and production [12,13,14].

Recent developments in metasurfaces have significantly advanced the control of electromagnetic waves. For instance, time-varying metasurfaces have shown great potential in applications such as radar stealth and ultra-wideband wireless communication [15]. Similarly, reconfigurable metasurfaces that manipulate amplitude, phase, and polarization have been demonstrated, offering a broad range of functionalities [16]. Additionally, the use of Pancharatnam–Berry phase elements in metasurfaces has been proven to maximize cross-polarization efficiency, providing further insights into polarization control [17]. These studies highlight the diverse capabilities of metasurfaces and provide a strong foundation for our investigation into polarization conversion mechanisms.

Prior studies have shown that metasurface-based polarization converters can be realized in both reflective and transmissive configurations [18,19]. Gao et al. enhanced mutual coupling in a low-loss Mylar substrate metasurface by reducing the distance between adjacent units, achieving a cross-polarization conversion ratio exceeding 0.8 in the 0.2–0.4 THz range [20]. Similarly, Grady and co-authors reported a terahertz polarization converter using metallic cut wires, obtaining conversion efficiencies of over 80% [21]. Karamirad et al. presented a broadband, ultra-thin polarization-rotating surface based on elliptical directivity with a polarization conversion ratio (PCR) greater than 90% [22]. In another study, Xu et al. developed linear polarization converters by arranging 3D open-ring resonators orthogonally, controlling efficiency via impedance tuning at common terminal ports [23]. Zhang’s group proposed an asymmetric cross-shaped polarization converter achieving over 80% linear polarization conversion within the 8.3–14.3 GHz range [24], while Cong Fang introduced a trapezoidal metasurface structure and elucidated an efficient reflective polarization conversion mechanism based on surface current distributions and destructive interference [25]. Lon Schmidcar and colleagues designed a reflective polarization converter [26], followed by a broadband converter incorporating open-ring resonators [27], and later a wideband polarizer employing high-impedance surfaces [28].

Departing from prior investigations, Xiaojun Huang has introduced a novel low-profile unit based on the reflective mode, achieving conversion from linear to circular polarization over two separate frequency bands [29]. For the ultra-broadband metasurface unit-cell structure, the most commonly used design approaches are designing single resonators with multiple resonant frequency points independently adjusting the unit structures to extend the bandwidth [30,31] and combining different types of resonators to induce additional mutual coupling effects within the device, thereby stitching two polarization conversion bands together [32,33]. However, existing studies often face limitations such as narrow bandwidth and low polarization conversion rates. Proposing a simple and reliable design for an ultrawideband polarization conversion metasurface that covers multiple frequency bands is an urgent issue in current research directions.

In this work, we propose an anisotropic ultrawideband polarization conversion metasurface (PCM) capable of converting incident linearly polarized electromagnetic waves into orthogonal polarization states over a wide frequency range (7.1–22.3 GHz), covering the C-, X-, Ku-, and K-bands. The proposed converter features double V-shaped metal patterns combined with circular split-ring resonators mounted on an F4B dielectric substrate with a metallic ground, thus forming a reflective metasurface. Our design achieves a PCR exceeding 90% across 7.1–22.3 GHz, yielding a relative bandwidth of 103% and a wide 15 GHz conversion band, significantly satisfying ultrawideband application requirements. An in-depth analysis of the physical mechanisms, including polarization conversion and multiple resonances induced by surface current distributions and Fabry–Pérot cavity effects, reveals the underlying working principles. Numerical simulations and experimental measurements exhibit consistent agreement, validating the performance of our design. Additionally, by arranging the PCM and its mirror image in a chessboard pattern, we realize a monostatic RCS reduction exceeding 10 dB across 7.6–21.5 GHz, underscoring its excellent electromagnetic stealth capabilities. These findings highlight the potential of polarization conversion metasurfaces for practical applications in radar systems and wireless communications, offering new insights for the radar field and beyond.

## 2. Materials and Methods

### 2.1. Design of the PCM

**Figure 1:**
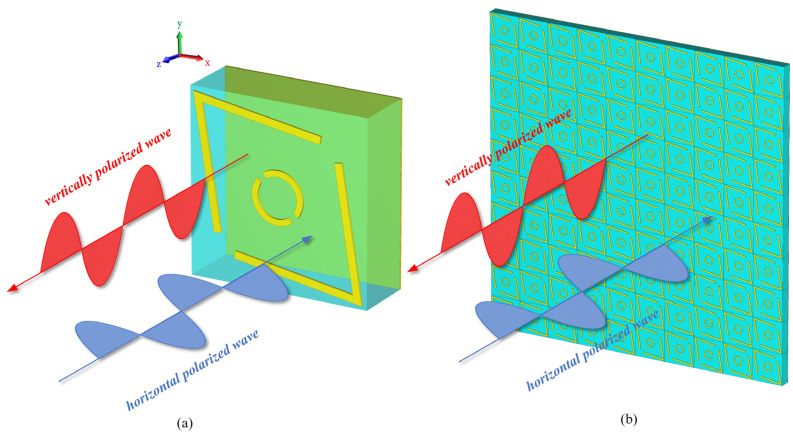
Schematic of the polarization converter: (**a**) Three-dimensional view of the PCM unit cell and (**b**) 10 × 10 PCM unit cell.

The 3D diagram of the PCM unit cell proposed in this paper is shown in Figure 1a. The design of the PCM unit structure consists of a three-layer configuration. Both the top and bottom metal layers are made of copper with an electrical conductivity σ of 5.8 × 10^7^ S/m. The top layer features a resonant structure composed of two symmetric “V” shapes embedding a double-split ring pattern, while the bottom layer is a fully covered metal ground layer. The middle layer is an F4B dielectric substrate with a relative permittivity ε_r_ of 2.1 and a loss tangent tanδ of 0.0002. The structure of the polarization converter is formed by a periodic array of anisotropic metasurfaces, as shown in Figure 1b.

**Figure 2:**
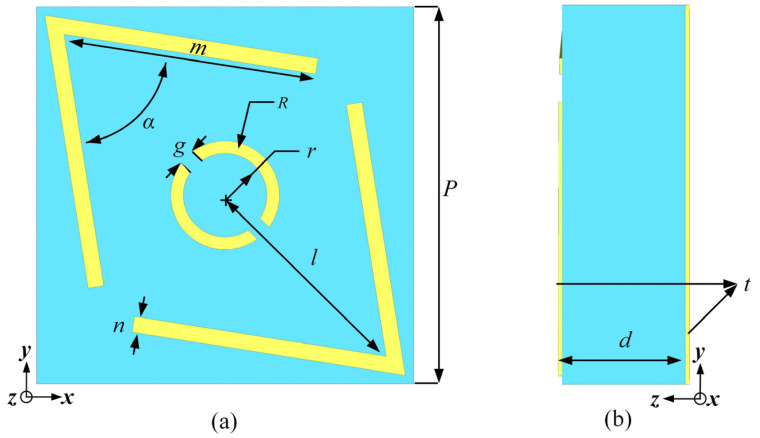
Proposed polarization converter unit cell: (**a**) front view and (**b**) side view.

Through simulation and optimization with CST Studio Suite 2023 (Release Version 2023.03—Feb 23 2023), the top and side views of the unit structure are shown in Figure 2. The final selected geometrical dimensions are as follows: the dielectric substrate is F4B with a thickness of 3.5 mm, and the thickness of the copper layers on the top and bottom is 0.017 mm. The periodicity of the unit structure is p = 9 mm. The other specific parameters are as follows: *m* = 6.6 mm, *n* = 0.28 mm, *l* = 4.3 mm, *g* = 0.2 mm, *d* = 3.5 mm, *t* = 0.017 mm, *α* = 75°, *R* = 0.8 mm, and *r* = 0.6 mm. The periodicity p is approximately 1/2 λ (λ represents the wavelength corresponding to the center frequency of 16.7 GHz.), and the length of the “V” shape m is approximately 1/3 λ.

### 2.2. Simulation Results

**Figure 3:**
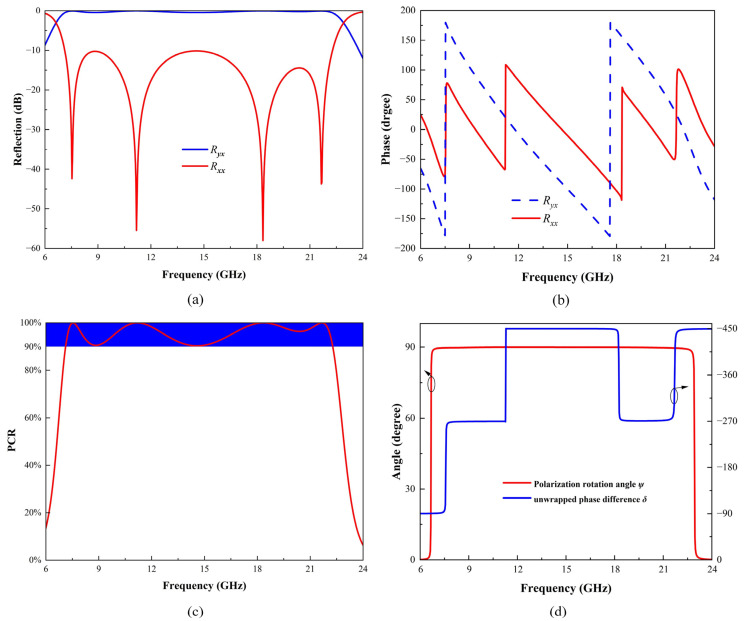
Reflection simulation results of this converter under normal x-polarization. (**a**) Amplitude and (**b**) phase of co- and cross-polarized reflection coefficients, (**c**) PCR, (**d**) polarization rotation angle ψ, and unwrapped phase difference δ for x-polarized normal incidence.

Full-wave simulations were conducted in CST Microwave Studio to verify the ultra-wideband polarization conversion capability of the proposed PCM. Periodic boundary conditions were set in the x and y directions as “unit cell”, while in the −z direction, an “electric (Et = 0)” boundary condition (perfect electric boundary) was set, and in the +z direction, an “open (add space)” boundary condition was used. The Floquet port mode was employed in the z-direction, which effectively extends a single PCM unit into an infinitely large periodic array structure along the x and y directions. Taking x-polarized incidence as an example, Figure 3a shows the amplitude of simulated cross-polarized and co-polarized reflection coefficients across the 6–24 GHz frequency range. Notably, within the 7.1–22.3 GHz range, the values of *R_yx_* exceed −0.05 dB, while those of *R_xx_* are less than −10 dB. Consequently, when x-polarized electromagnetic waves impinge on the surface of the PCM structure, they are converted into y-polarized reflected waves, thereby facilitating linear polarization conversion. Importantly, the ultrawideband polarization conversion characteristic is extended by adjacent resonant frequencies at 7.525 GHz, 11.179 GHz, 18.342 GHz, and 21.648 GHz, whose proximity generates a broadband effect. At these four resonant points, their cross-polarized reflection coefficients are −45 dB, −62 dB, −67 dB, and −42 dB, respectively, indicating that the polarization conversion rates are close to 100% at these resonant points.

Figure 3b illustrates the phases of *R_yx_* and *R_xx_* within the corresponding frequency range, where the phase difference between them is approximately ±90°. Notably, abrupt phase changes occur at certain frequencies, specifically at 7.525 GHz, 11.179 GHz, 18.342 GHz, and 21.648 GHz, which correspond to the resonant points of the structure. These sudden variations are caused by strong coupling and rapid phase transitions inherent to multi-resonant behavior, which are consistent with phenomena observed in over-coupled metasurface designs [34]. Such phenomena are typical in metasurfaces designed for broadband polarization conversion, where the presence of closely spaced resonant modes induces significant phase shifts across the operating frequency range.

Figure 3c illustrates the PCR results across the target operating frequency range. To assess the efficiency of the polarization conversion, we introduce the PCR. Assuming the incident wave is x-polarized and propagates along the x-axis, the PCR is expressed as Equation (1). The following criteria for calculating PCR, as applied in this study, have been extensively discussed in the literature [35]:

(1) $$\mathrm{PCR}={\left|R_{yx}\right|}^{2}/({\left|R_{yx}\right|}^{2}+{\left|R_{xx}\right|}^{2})$$

The PCR quantifies the efficiency of polarization conversion, with higher values indicating a more effective conversion of the incident wave’s polarization state. Apparently, the calculated PCR exceeds 90% over the range of 7.1–22.3 GHz. Fractional bandwidth is a measure used to describe the width of the bandwidth relative to the center frequency, with the following calculation formula represented as Equation (2):

(2) $$B_{f}=2\times (f_{h}-f_{l})/(f_{h}+f_{l})$$

The highest and lowest frequencies of the operational bandwidth with a specific conversion efficiency are denoted as *f_h_* and *f_l_*, respectively. After calculations, the fractional bandwidth for polarization conversion of the PCM presented in this paper is 103%, substantially exceeding the requirements for ultrawideband applications.

In order to accurately describe the case of electromagnetic waves undergoing PCM polarization conversion, we consider an incident X-polarized wave as an example and introduce the Stokes method to determine the polarization state using four parameters of the same physical dimension [36], as shown in Equations (3)–(7) following:

(3) $$S_{0}=R_{xx}^{2}+R_{yx}^{2}$$

(4) $$S_{1}=R_{xx}^{2}-R_{yx}^{2}$$

(5) $$S_{2}=2R_{xx}R_{yx}\cos\delta$$

(6) $$S_{3}=2R_{xx}R_{yx}\sin\delta$$

(7) $$\sin2\psi =S_{2}/S_{1}$$

In this context, represents the phase difference between *R_xx_* and *R_yx_*, while *ψ* is the polarization rotation angle for linear polarization conversion, as shown in Figure 3d. Within the 7.1 to 22.3 GHz range, the polarization rotation angle ψ is 90 degrees, indicating that the polarization converter can transform x-polarized incident waves into almost purely y-polarized waves across a broad bandwidth.

**Figure 4:**
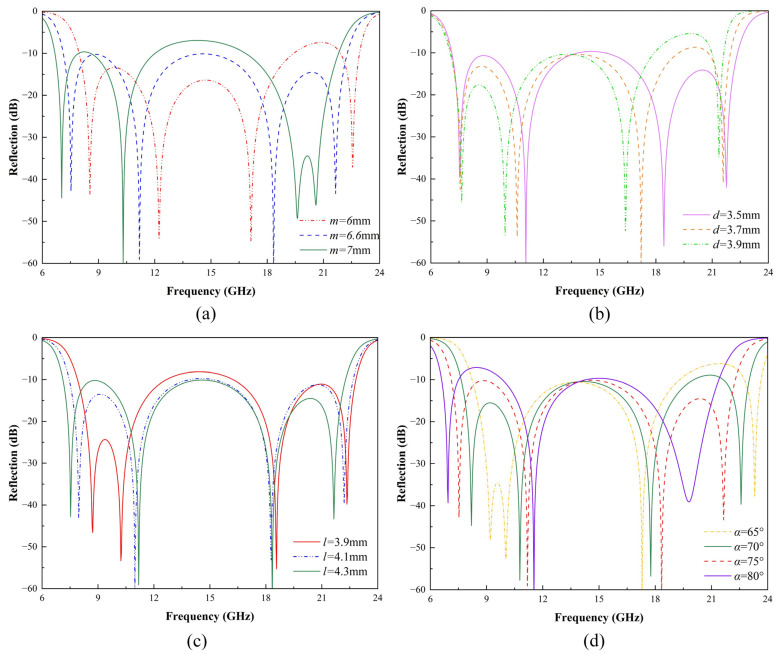
Effects of geometric parameters on the co-polarized reflection coefficient: (**a**) V-shaped arm length *m*, (**b**) substrate thickness *d*, (**c**) distance from the V-shaped structure to the center *l*, and (**d**) opening angle α.

To better understand the design concept and investigate the influence of geometric parameters on the resonance characteristics of the proposed metasurface under x-polarized linearly polarized waves, a comprehensive parametric analysis was conducted. The effects of varying the V-shaped arm length *m*, substrate thickness *d*, distance from the V-shaped structure to the center *l*, and opening angle *α* on the co-polarized reflection coefficient are illustrated in Figure 4.

Figure 4a shows that increasing the V-shaped arm length *m* shifts the first and second resonance points to lower frequencies. Meanwhile, the third and fourth resonance points move closer to each other, exhibiting a trend of merging. As shown in Figure 4b, variations in substrate thickness *d* affect the electromagnetic coupling between the resonators and the ground plane. A thicker substrate increases the effective capacitance, resulting in a downward frequency shift, particularly noticeable in the mid-frequency resonances. In Figure 4c, the distance between the V-shaped structure and the center *l* is varied. Increasing *l* primarily affects the first and second resonance points, causing the first resonance to shift to higher frequencies and the second to shift to lower frequencies, thereby bringing the two resonance points closer together. Figure 4d shows the effect of the opening angle *α*. Different opening angles significantly influence the first and fourth resonances. As the opening angle increases, the first resonance shifts closer to the second, forming a stronger combined resonance. Similarly, the fourth resonance moves toward lower frequencies and merges with the third resonance, resulting in a more pronounced resonance.

The parametric analysis reveals that the four observed co-polarization spectral dips are highly sensitive to changes in the unit cell geometry. These findings highlight the importance of precise geometrical tuning to achieve the desired broadband polarization conversion performance.

## 3. Theoretical Analysis and Discussion

### 3.1. Physical Mechanisms Analysis

**Figure 5:**
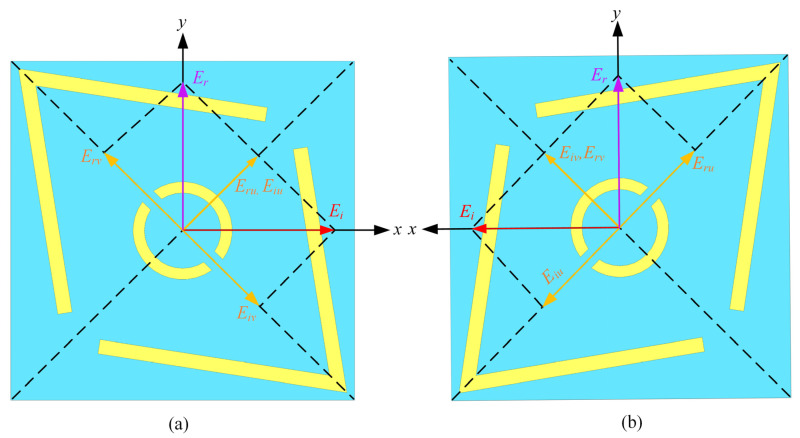
(**a**) The proposed unit cell for polarization conversion incorporating electric field decomposition and (**b**) mirrored the proposed polarization converter unit cell.

To further elucidate the basic physical mechanisms of the proposed metasurface when subjected to X-polarized incident waves, the polarization state is decomposed into two orthogonal components, u and v, obtained by rotating the x-axis and y-axis counterclockwise by 45°, respectively, as illustrated in Figure 5. The incoming field is Equation (8), and the reflected field is Equation (9), which represents the reflection coefficients along the u and v axes, respectively [22]. Owing to the metasurface’s anisotropic nature, a phase disparity (Δφ) arises between ${\vec{R}}_{uu}$ and ${\vec{R}}_{vv}$ as follows:

(8) $${\vec{E}}_{i}=\hat{u}E_{iu}e^{\;j\phi }+\hat{v}E_{iv}e^{\;j\phi }$$

(9) $${\vec{E}}_{r}=\hat{u}E_{ru}e^{\;j\phi }+\hat{v}E_{rv}e^{\;j\phi }=\hat{u}R_{uu}E_{iu}e^{\;j\phi }\;e^{\;j\phi_{u}}+\hat{v}R_{vv}E_{iv}e^{\;j\phi_{v}}$$

In Figure 5a, the composite field of ${\vec{E}}_{ru}$ and ${\vec{E}}_{rv}$ is obtained along the x-direction, under the condition $R_{uu}\approx R_{vv}$ and $\Delta \varphi =\left|\varphi_{u}-\varphi_{v}\right|\approx 180^{{}^{\circ}}$. Thus, the reflected electromagnetic field attains a 90° polarization rotation. The corresponding coefficients of reflection are expressed as $R_{yx}=\left|E_{yr}/E_{xi\;}\right|$ and $R_{xx}=\left|E_{xr}/E_{xi\;}\right|$, where “$E_{xi\;}$” indicates the y-polarized incident electric field. Furthermore, “R” represents the reflection coefficients for x-to-y and x-to-x polarization rotation. Figure 5b shows the mirrored structure of the proposed PCM unit cell. Due to symmetry, the decomposed electric fields in relation to the PCM will differ only by a phase shift of 180 degrees. To substantiate the polarization rotation described above, numerical simulations of the reflection coefficients of the metasurface under u-axis and v-axis polarized illumination were conducted.

**Figure 6:**
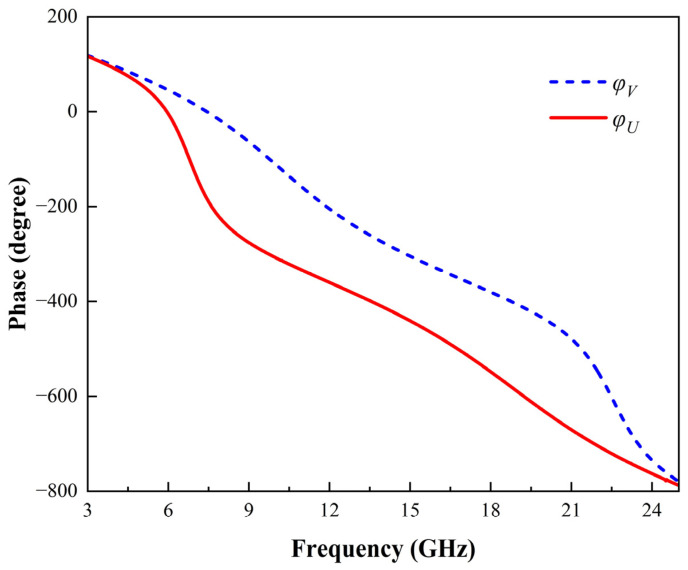
Unwrapped phase difference under normal incidence in the UV direction. The magnitude remains above 97% in the band of interest.

The unwrapped phase difference under normal incidence in the UV direction over the 3–25 GHz frequency range is presented in Figure 6. As shown, the phase difference between the two reflected components remains close to 180° within the 7–22 GHz band, which is essential for achieving efficient polarization conversion. This stable phase relationship ensures that the incident linearly polarized waves are effectively converted into orthogonal polarization states over a broad frequency range. Additionally, the reflection magnitude exceeds 97% throughout this band, guaranteeing minimal energy loss during the polarization conversion process.

The combination of a stable phase difference near 180° across the 7–22 GHz band and a high reflection magnitude enables highly efficient polarization conversion over the target frequency range.

### 3.2. Surface Current Distributions

**Figure 7:**
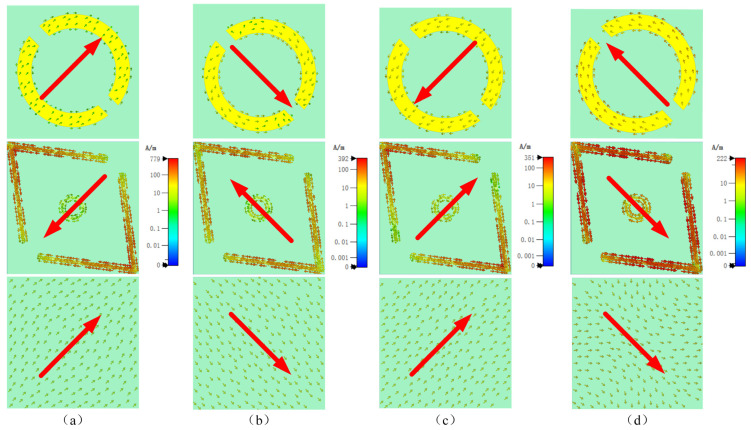
Surface current (A/m) profiles of the metallic components and the ground plane in the proposed unit cell for a wave normally incident along the x-axis at frequencies of (**a**) 7.525 GHz, (**b**) 11.179 GHz, (**c**) 18.342 GHz, and (**d**) 21.648 GHz.

To further explore the physical mechanism of the ultra-wideband polarization conversion of the proposed PCM, we analyze the surface current distributions [22,37] on the top artificial electromagnetic structure and the bottom full-metal copper-covered backplane of the unit at four resonant frequencies (7.525 GHz, 11.179 GHz, 18.342 GHz, and 21.648 GHz). Field monitors are added at these four resonant points. The incident electromagnetic wave is a linearly polarized wave in the x-direction, and the surface current distributions on the top and bottom surfaces at each resonant point are shown in Figure 7a–d, with the main direction of the surface currents represented by red arrows.

At 7.525 GHz, Figure 7a shows an opposite current flow between the V-shaped top layer and the bottom layer, suggesting a magnetic dipole resonance as per Faraday’s law. This resonance, enhanced by an increased magnetic permeability μ, leads to a higher impedance surface ($\eta =\sqrt{\mu }/\varepsilon$), resulting in in-phase reflection. Additionally, the alignment of some currents on the annular top layer with the induced bottom layer currents initiates an electric dipole resonance. Figure 7b reveals that at 11.179 GHz, the antipodal current flow between the layers leads to the formation of a magnetic dipole resonance. At 18.342 GHz, Figure 7c demonstrates aligned currents on both layers, indicative of an electric dipole resonance stimulation. Conversely, opposing currents on the ring and the bottom layer’s edge at the same frequency instigate a magnetic dipole resonance. Lastly, at 21.648 GHz, Figure 7d shows aligned currents on both layers, exciting an electric dipole resonance. The presence of contrary-induced currents on both layers further supports this resonance. Through the mutual coupling between the inner and outer regions of different resonator patterns, four resonant points are generated, each distributed in adjacent frequency bands. These points are interconnected, effectively stitching the polarization conversion bands together, thereby expanding the bandwidth. This approach successfully achieves high-efficiency ultra-wideband polarization conversion within the 7.1–22.3 GHz range.

## 4. RCS Reduction

Artificial Magnetic Conductors (AMCs) are engineered electromagnetic structures that exhibit high-impedance surfaces within specific frequency ranges. This characteristic results in a reflection coefficient of +1 for normally incident electromagnetic waves, meaning the reflected wave is in phase with the incident wave, analogous to an open-circuit condition in transmission lines. In contrast, Perfect Electric Conductors (PECs) have zero impedance surfaces, yielding a reflection coefficient of −1, where the reflected wave is 180 degrees out of phase with the incident wave, similar to a short-circuit condition. The unique in-phase reflection property of AMCs makes them advantageous in applications such as reducing back radiation and enhancing antenna gain.

Due to the frequency-dependent phase variation of AMCs, the two selected AMC structures must satisfy a phase difference of 180° ± 37° within a certain bandwidth. By arranging these two AMC structures in a chessboard pattern, destructive interference between the reflected waves can be achieved, which expands the RCS reduction bandwidth. To simplify the design, a single PCM structure is proposed to replace the two different AMC structures, achieving the chessboard pattern with the same unit. As shown in Figure 5b, according to the principle of polarization conversion, rotating the same PCM structure by 90° around its center can create a 180° phase difference between it and the original structure. When a chessboard-like structure is formed using the PCM unit and its mirror unit, the polarization conversion rate of the PCM itself becomes the only factor affecting the RCS reduction performance. Assuming that the incident wave is an x-polarized electromagnetic wave, it is fully reflected and completely transformed into a y-polarized reflected wave orthogonal to the incident wave after passing through the PCM surface. Since the reflected waves generated by the PCM unit and its mirror unit will undergo destructive interference, the energy of the reflected waves is weakened. Therefore, arranging the PCM units and their mirror units in a chessboard-like structure can effectively achieve RCS reduction.

**Figure 8:**
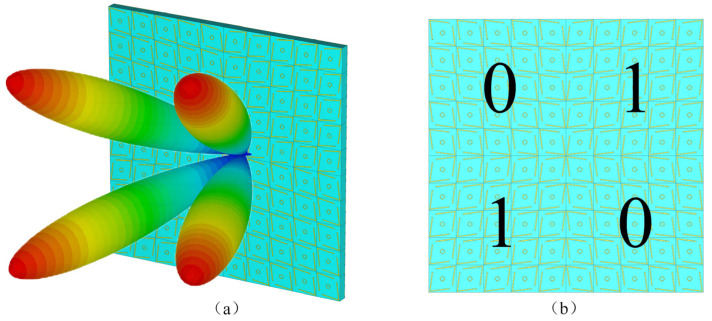
PCM unit cell chessboard structure for RCS reduction: (**a**) Three-dimensional schematic and (**b**) front view.

As shown in Figure 8, the schematic diagram of the scattering suppression principle for the chessboard-type PCM is presented. This PCM consists of two subarrays, Subarray 0 and Subarray 1, arranged in an interleaved manner. Subarray 0 and Subarray 1 are arrays of polarization converters and their mirror image structures, respectively. When the electromagnetic wave is incident vertically onto the PCM, the total scattered field is the superposition of the scattered fields from each individual unit cell. The scattered fields from the two types of unit cells can be defined [38] as follows:

(10) $${\vec{E}}_{1}=A_{1}e^{j\varphi_{1}}\cdot \hat{z},\;{\vec{E}}_{2}=A_{2}e^{j\varphi_{2}}\cdot \hat{z}$$

Here, ${\vec{E}}_{1}$ and ${\vec{E}}_{2}$ represent the reflected electric fields of Unit 0 and Unit 1, respectively. The terms $A_{1}$, $A_{2}$, $\varphi_{1}$, and $\varphi_{2}$ denote the reflection amplitudes and reflection phases of the two unit cells. The total scattered field of the PCM can be expressed as follows:

(11) $${\vec{E}}_{\mathrm{sca}}=A_{1}e^{j\varphi_{1}}\cdot AF_{1}\cdot \hat{z}\;+\;A_{2}e^{j\varphi_{2}}\cdot AF_{2}\cdot \hat{z}$$

The array factors of Subarray 0 and Subarray 1, denoted as $AF_{1}$ and $AF_{2}$, are given by the following:

(12) $$\begin{array}{l} AF_{1}=[e^{j\frac{k_{x}d_{x}+k_{y}d_{y}}{2}}\;+\;e^{j\frac{-k_{x}d_{x}-k_{y}d_{y}}{2}}]\\ AF_{2}=[e^{j\frac{-k_{x}d_{x}+k_{y}d_{y}}{2}}\;+\;e^{j\frac{k_{x}d_{x}-k_{y}d_{y}}{2}}] \end{array}$$

Here, *d_x_* and *d_y_* represent the distances between the centers of the two subarrays along the x- and y-directions, respectively. The wave numbers along the x- and y-axes are given by the following: $k_{x}=2\pi \sin\theta \cos\varphi /\lambda$, $k_{y}=2\pi \sin\theta \sin\varphi /\lambda$. By combining the radar cross-section (RCS) calculation formula, the scattered field of the target can be expressed as follows:

(13) $$\sigma =\underset{R\to \infty }{\lim}\;4\pi R^{2}\left(\frac{{\left|{\vec{E}}_{\mathrm{sca}}\right|}^{2}}{{\left|{\vec{E}}_{\mathrm{inc}}\right|}^{2}}\right)$$

Here, ${\vec{E}}_{\mathrm{sca}}$ and ${\vec{E}}_{\mathrm{inc}}$ represent the scattered field and the incident field, respectively. Therefore, the RCS reduction capability of the PCM, compared to a metal plate of the same size, can be expressed as follows:

(14) $${\mathrm{RCS}}_{\mathrm{reduction}}=10\log_{10}[\frac{\underset{R\to \infty }{\lim}4\pi R^{2}\left(\frac{{\left|{\vec{E}}_{\mathrm{sca}}\right|}^{2}}{{\left|{\vec{E}}_{\mathrm{inc}}\right|}^{2}}\right)}{\underset{R\to \infty }{\lim}4\pi R^{2}{\left(1\right)}^{2}}]=10\log_{10}[\frac{{\left|{\vec{E}}_{\mathrm{sca}}\right|}^{2}}{{\left|{\vec{E}}_{\mathrm{inc}}\right|}^{2}}]$$

For the chessboard-type PCM, neglecting dielectric and conductor losses yields $A_{1}$ = $A_{2}$ = 1. Therefore, the RCS reduction capability of the PCM under normal incidence of electromagnetic waves can be expressed as follows:

(15) $$\begin{array}{l} {\mathrm{RCS}}_{\mathrm{reduction}}=20\log_{10}|\frac{e^{j\varphi_{1}}+e^{j\varphi_{2}}}{2}|<-10\\ |e^{j\varphi_{1}}+e^{j\varphi_{2}}{|}^{2}=2+2\cos(\varphi_{1}-\varphi_{2})\leq 0.4\\ \to \cos(\varphi_{1}-\varphi_{2})\leq -0.8\\ ↔143^{{}^{\circ}}\leq |\varphi_{1}-\varphi_{2}|\leq 217^{{}^{\circ}}\\ ↔|\varphi_{1}-\varphi_{2}|\leq 180^{{}^{\circ}}\pm 37^{{}^{\circ}} \end{array}$$

Based on the derivation of the equations, when the reflection phase difference between the two types of unit cells forming the chessboard-type metasurface lies within the range of 180° ± 37°, the metasurface can achieve an RCS reduction exceeding 10 dB compared to a metal plate of the same size.

In this paper, an ultra-wideband PCM chessboard structure is designed using the previously proposed ultra-wideband polarization conversion unit, achieving an RCS reduction of more than 10 dB over an ultra-wideband range. This unit features advantages such as a simple structure, lightweight thickness, ultra-wide polarization conversion bandwidth, and high polarization conversion rate. As shown in Figure 8, through simulation optimization, the chessboard structure is chosen to be composed of 10 × 10 units. An equal-sized bare F4B board with full copper backing is selected as the reference.

**Figure 9:**
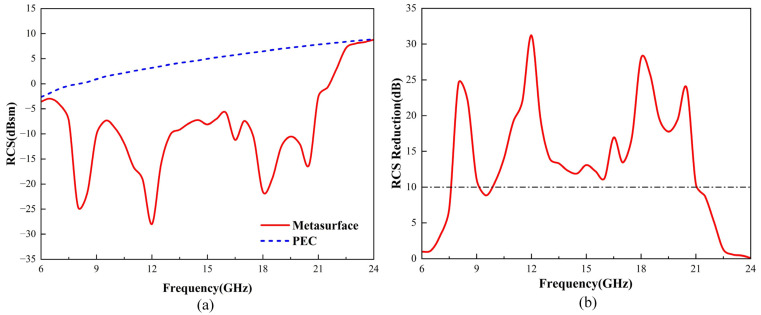
Electromagnetic wave vertical incidence simulated via CST Microwave Studio: (**a**) Comparison of RCS between the PCM chessboard structure and a metal plate of the same size and (**b**) RCS reduction curve.

The simulation was carried out by CST software; an x-polarized electromagnetic wave is vertically incident on the encoded metasurface and a metal plate of the same size, and the RCS distribution in the far field is simulated. Figure 9a presents the RCS distribution of the PCM unit cell chessboard arrangement compared to a metal plate of the same size within the 6–24 GHz range, while Figure 9b shows the RCS reduction curve of the metasurface relative to the same-sized metal plate as a function of frequency. It can be seen that the designed metasurface has good RCS reduction capability in the frequency range of 7.6–21.5 GHz. Except for the 9–10 GHz frequency band, where the RCS reduction value is slightly less than 10 dB, with the minimum reduction being 8.9 dB, the RCS reduction values at other frequency points are all greater than 10 dB. The RCS of the metasurface has minimum values at frequency points of 8 GHz, 12 GHz, 18 GHz, and 20.5 GHz, with corresponding reduction amounts of 24.4 dB, 31.2 dB, 28.1 dB, and 23.8 dB, respectively. By comparing Figure 3c and Figure 9b, it can be found that the curve of the polarization conversion rate of the unit structure varying with frequency is roughly consistent with the curve of the RCS reduction of the metasurface varying with frequency. This is because, at frequency points with high polarization conversion rates, the phase difference in the response of the two-unit structures to the incident wave tends to approach 180°, resulting in better phase control of the metasurface on the incident wave. Conversely, at frequency points with low polarization conversion rates, the phase difference in the response of the two-unit structures to the incident wave tends to approach 0°, resulting in poorer phase control of the metasurface on the incident.

**Figure 10:**
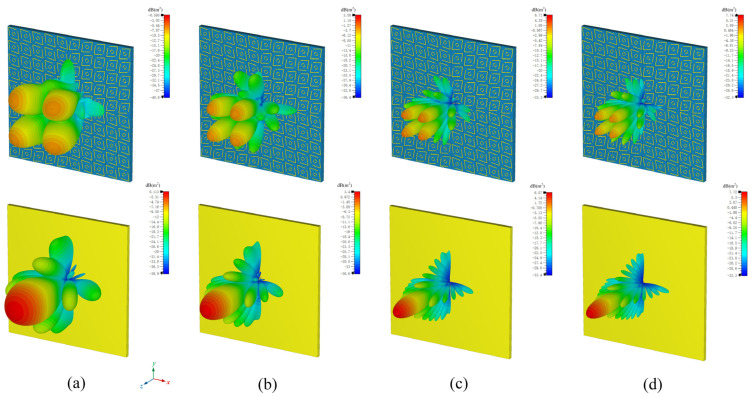
Three-dimensional RCS patterns at different frequencies: (**a**) 8 GHz, (**b**) 12 GHz, (**c**) 18 GHz, and (**d**) 20.5 GHz.

For a more intuitive understanding, Figure 10a–d, respectively, present the three-dimensional scattering field comparisons of the metasurface chessboard structure PCM with a metal plate of the same size at frequencies of 8, 12, 18, and 20.5 GHz. When electromagnetic waves are incident perpendicularly, the diagrams clearly show a significant reduction in RCS in the vertical direction for the metasurface compared to the reference metal plate, divided into four distinct reflective sidelobes. In other words, this PCM chessboard structure effectively redirects the energy of the backward main lobe into other non-vertical directions, thereby substantially reducing the RCS near the vertical direction. A comparison of these images reveals higher sidelobe energy within the metasurface, indicating that the main lobe energy is diminished, leading to effective scattering and a notable capability to reduce RCS.

## 5. Experimental Verification and Comparative Analysis

**Figure 11:**
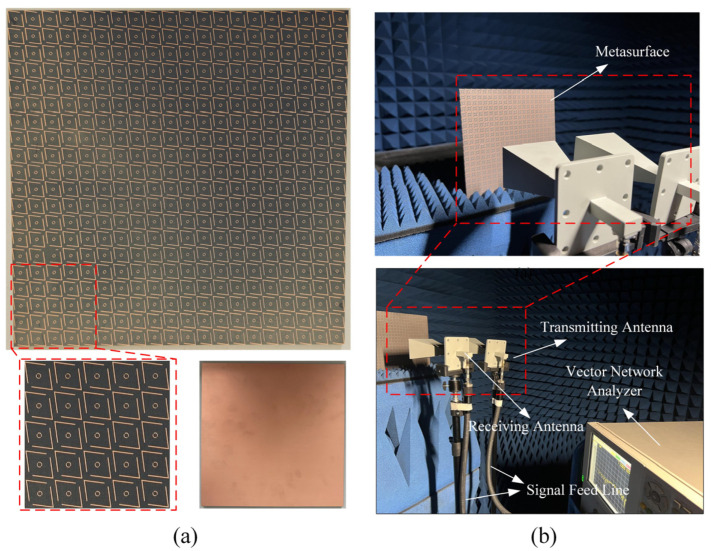
Fabricated prototype and measurement environment. (**a**) Fabricated prototype of 20 × 20 array of proposed PCM and (**b**) photograph of the PCM measurement setup.

To verify the performance of the polarization converter proposed in this paper, we fabricated a PCM structure with a total size of 18 × 18 cm^2^, consisting of 20 × 20 unit cells, as shown in Figure 11a. The experimental setup is illustrated in Figure 11b, where the reflection coefficients of the metasurface were tested using VNA and horn antennas. Two identical horn antennas were connected to the two ports of an Agilent E5230A VNA (sourced from Agilent Technologies, Santa Clara, California, USA) via signal feedlines. Due to the extensive frequency range of the tests, two sets of horn antennas covering different frequency bands (2–18 GHz and 18–27 GHz) were used.

**Figure 12:**
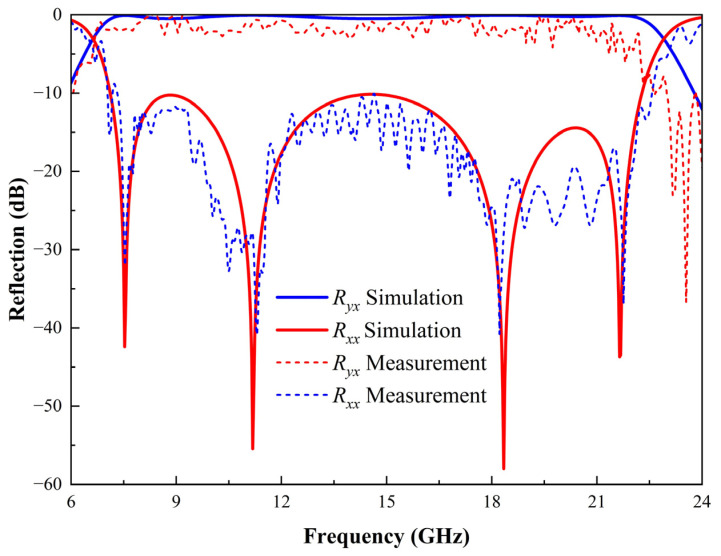
Comparison of reflection simulation results and experimental measurements of the PCM.

Initially, frequency and S-parameter calibration were conducted using the VNA, alongside calibration of the horn antenna’s gain. Both antennas were positioned in the same orientation (either vertical or horizontal), and the co-polarized reflection coefficients *R_xx_* or *R_yy_* were obtained from the S21 parameter of the VNA. Subsequently, by rotating the horn antenna connected to Port 2, the cross-polarized reflection coefficients *R_yx_* or *R_xy_* could be determined from the current S21 parameter of the VNA. Notably, to compensate for the microwave link losses between the transmitting and receiving antennas, the actual co-polarized and cross-polarized reflection coefficients were derived by subtracting the S21 parameter measurements of a normal metal surface of the same dimensions. Based on the measured S-parameter data, the frequency response curves were plotted as shown in Figure 12. The selected F4B dielectric exhibited increasing loss with frequency. Nevertheless, four resonance points were clearly observed at 7.525, 11.179, 18.342, and 21.648 GHz. As evident from the graph, the simulation data closely align with the experimental measurements. These findings robustly substantiate the reciprocal polarization transformation between x-polarized and y-polarized electromagnetic waves across an extensive frequency range.

**Figure 13:**
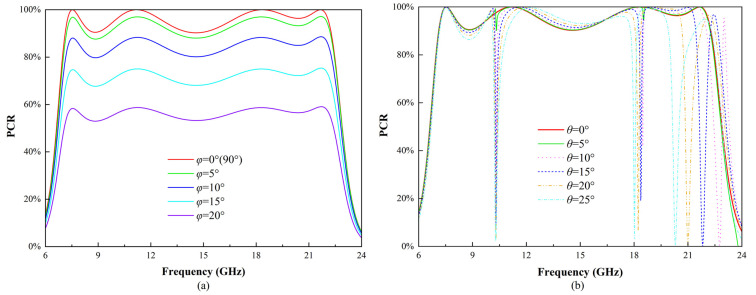
Simulated PCR via CST Microwave Studio under various incident conditions. The results for x- and y-polarized incidences are identical, indicating polarization insensitivity along the principal axes. (**a**) PCR at different polarization angles under normal incidence and (**b**) PCR under oblique incidence with varying angles.

To evaluate the polarization stability of the proposed metasurface, the PCR was simulated under various polarization angles *φ*, as shown in Figure 13a. The results indicate that for x- and y-polarized incidences (*φ* = 0° and *φ* = 90°), the PCR curves overlap, demonstrating polarization insensitivity along the principal axes.

However, as the polarization angle deviates from the principal axes, the PCR exhibits a notable decline. When the polarization angle is within 10° of the principal axes, the PCR remains above 80% across most of the operating frequency range (7–22 GHz), ensuring stable performance under slight misalignments. In contrast, at a polarization angle of 20°, the PCR decreases to approximately 50%, indicating a more significant impact as the incident polarization direction deviates further from the principal axes. This behavior stems from the anisotropic nature of the metasurface unit cell, where polarization conversion efficiency is maximized when the incident electric field aligns with the principal axes but becomes less effective as the angle deviates.

Figure 13b presents the simulated polarization conversion ratio (PCR) of the proposed metasurface under various oblique incidence angles *θ* to evaluate its angular stability. For incident angles up to 15°, the PCR remains above 90% across most of the operating frequency range (7–22 GHz), with only a few isolated frequency points showing slight deviations. As the angle increases beyond 15°, the effective bandwidth gradually decreases; however, high polarization conversion efficiency is still maintained. This performance decline at higher angles is primarily attributed to variations in wave propagation paths and reduced resonant mode excitation efficiency. Overall, the metasurface demonstrates excellent angular stability, ensuring robust and efficient polarization conversion over a wide frequency range under oblique incidence.

Finally, Table 1 provides a comparison between the work proposed and existing technical references. It can be seen from the table that, compared to the literature reviewed, the proposed polarization converter features a higher relative bandwidth and a much simpler structural design. In the comparative literature, broadband was achieved through techniques such as through-hole connections, multilayer structures, or cover layers. It is noteworthy that the proposed metasurface was realized on a low-cost, lightweight commercial F4B substrate, which facilitates its application. Moreover, unlike other references, this paper also introduces a radar cross-section (RCS) reduction design, expanding the potential applications for the PCM.

## 6. Conclusions

In this study, we propose an efficient, low-cost anisotropic ultra-wideband cross-polarization converter and an ultra-wideband RCS reduction scheme. This polarization converter spans the C, X, Ku, and K frequency bands with co-polarized conversion resonant frequencies at 7.525, 11.179, 18.342, and 21.648 GHz, respectively. It effectively converts linearly polarized incident waves into orthogonally polarized reflected waves. The PCR of this converter exceeds 90% across a frequency range from 7.2 GHz to 22.71 GHz, achieving a fractional bandwidth of 103%. We discussed the physical mechanisms of the proposed PCM structure through analysis of surface currents, mathematical modeling, and UV phase changes. To validate the simulation results, we conducted experimental measurements that aligned with the numerical simulations. Due to its exceptional properties, our proposed PCM design holds substantial potential for applications in fields such as microwave communications, satellites, and radar. Furthermore, leveraging the structure of this polarization converter, we designed an ultra-wideband RCS reduction metasurface. This chessboard-like structure achieves an RCS reduction of over 10 dB in the range of 7 to 21 GHz, with a maximum reduction reaching 30 dB, making it suitable for applications in radar electromagnetic stealth.

## Figures and Tables

**Table 1:** Performance comparison of the proposed metasurface with other polarization converters.

Ref	d/λ_1_	OB (GHz)	OBWb(GHz)	RB (%)
[22]	0.068	10.2–20.5	10.3	67%
[39]	0.066	9.04–20.83	11.79	79%
[40]	0.071	17.97–40.23	22.26	76.5%
[41]	0.042	8–12	4	40%
[42]	0.11	5.66–9.46 16.9–18.9	3.8	50%
[33]	0.077	6.67–17.1	10.43	87.7%
[43]	0.05	7.5–8.6, 15–16.6	1.1, 1.6	13.7%, 10.1%
[44]	0.043	6.45–6.87, 9.94–11.34, 15.99–18.60	0.42, 1.4, 2.62	6.3%, 13.2%, 15.1%
[45]	0.064	12–18	6	40%
[46]	0.071	7.07–7.46, 16.59–16.91	0.39, 0.32	5.3%, 2%
[47]	0.103	15.5–16.5	1	6.3%
This Work	0.084	7.1–22.3	15.2	103.4%

d: the total thickness of the structure, λ1: lowest frequency of the band, OB: operating band (PCR > 90%), OWB: operating bandwidth (PCR > 90%), RB: relative band (PCR > 90%).

## Data Availability

The original contributions presented in this study are included in the article. Further inquiries can be directed to the corresponding author(s).
